# Nursing and Continuing Care Management Work Plan for People Living With COVID-19: Case Study of the Nakhon Pathom Province

**DOI:** 10.2196/65310

**Published:** 2025-05-29

**Authors:** Hathaichanok Buajaroen, Darin Photangtham, Wariya Chankham, Paisan Simalaotao, Ubonrat Sirisukpoca, Monchai Kongkamsook, Pantip Timtab, Tapanee Suasangei

**Affiliations:** 1 Community Nursing, Faculty of Nursing, Nakhon Pathom Rajabhat University, Nakhon Pathom, Thailand; 2 General Management Program, Faculty of Management Sciences, Nakhon Pathom Rajabhat University, Nakhon Pathom, Thailand; 3 Psychiatric and Mental Health Nursing, Faculty of Nursing, Nakhon Pathom Rajabhat University, 85 Maliaman Road, Nakhon Pathom, 73000, Thailand, 66 896611549; 4 Department of Computer Science, Faculty of Science and Technology, Nakhon Pathom Rajabhat University, Nakhon Pathom, Thailand; 5 Na-Sang, Subdistrict Health Promotion Hospital Health, Nakhon Pathom, Thailand; ^6^Faculty of Nursing, Nakhon Pathom Rajabhat University, Nakhon Pathom, Thailand

**Keywords:** post-COVID-19 symptoms, continuous care, database system, COVID-19, care, nursing, care management, Coronavirus, case study, needs, nurse, design, develop, database system, continuous monitoring, participatory research, Thailand, Asia, Asian, cough, fatigue, recovery, quality of care

## Abstract

**Background:**

Patients with post-COVID-19 continue to experience lingering physical and psychological symptoms, requiring coordinated and continuous care. Addressing these needs is essential, especially in resource-limited settings.

**Objective:**

The objectives of this paper are to study the issues and needs, as well as the nursing and continuous care systems for residents living with COVID-19, to design and develop a database system, develop continuous care guidelines, and evaluate the effectiveness of the database system for continuous monitoring and care for residents living with COVID-19 in Nakhon Pathom Province, Thailand.

**Methods:**

Participatory action research was used to engage stakeholders and guide the development process.

**Results:**

A total of 375 patients and family members affected by post-COVID-19 symptoms reported that symptoms persisted for approximately 6 months, with common symptoms including persistent cough and easy fatigue. These patients experienced reduced access to health care services, relying mainly on symptomatic treatment at local facilities and using telehealth nursing systems. They expressed a need for continuous care support from 50 professional nurses and village health volunteers. As a result, health care guidelines for post-COVID recovery were developed, comprising 5 core components: (1) self-care through digital information retrieval, (2) care via telehealth nursing systems, (3) physical health care services postrecovery, (4) mental health services postrecovery, and (5) continuous care for referral in case of postrecovery incidents. These guidelines were used to design a database system for continuous monitoring and care, which was evaluated as highly effective (mean 4.51, SD 0.59).

**Conclusions:**

This research highlights the critical need for a proactive and comprehensive approach to managing post-COVID-19 care in Nakhon Pathom Province. By developing and implementing a database system for continuous monitoring and care, along with clear guidelines, the study effectively addresses the ongoing needs of individuals recovering from COVID-19. The integration of technology, along with continuous care provided by professional nurses and village health volunteers, has been shown to be highly effective in improving the quality of care. The findings suggest that adopting these strategies, along with implementing supportive policies on data management and communication systems focused on home visits, will significantly enhance health service management and better prepare the region for future public health challenges.

## Introduction

### Background

As of October 1, 2022, the COVID-19 pandemic has transitioned from a global pandemic to an endemic disease. According to the latest data from the Department of Disease Control, the global number of confirmed cases from December 2019 to October 2021 reached 224,423,325, with 65,446 currently hospitalized and 4,963,653 cumulative deaths. In Thailand, from April 2021 to October 2022, there have been 4,660,878 cumulative cases, 2616 new cases, and 32,828 cumulative deaths. Specifically, in Nakhon Pathom Province, from April 2021 to October 2022, there have been 83,004 cumulative cases, 30 new cases, and 802 cumulative deaths [1,2]. These statistics show a declining trend in infections; however, there is still no clear report on the long-term effects on those who have recovered from COVID-19.

It is well known that the impact of the COVID-19 pandemic has caused rapid and severe shock, significantly affecting the global economy to the greatest extent in 150 years. The pandemic has impacted tourism for no less than 6 months, resulting in a loss of income exceeding 250 billion baht [3,4]. Despite the reopening of the country and the resumption of tourism, the economic recovery is still in progress and uncertain. The future trajectory of the pandemic and its long-term effects on those infected remain unclear.

Based on the experience in designing health service systems, nursing systems, and continuing care for at-risk populations and those infected with COVID-19 over the past 3‐4 years, the Faculty of Nursing at Nakhon Pathom Rajabhat University has found that the establishment of a database system to monitor the symptoms of at-risk individuals and patients with COVID-19 under the university’s rapid response system still lacks a comprehensive database system to support care within Local Quarantine Centers, Home Isolation, and Community Isolation.

Due to the spread of COVID-19, the health service system has not yet identified suitable methods for living and adapting to the ongoing presence of infectious diseases. There has been no planning or design of a system to access medical care and develop health care guidelines for COVID-19 patients to ensure they can correctly and safely manage their initial care. Additionally, there is no established guideline for postrecovery care for patients returning to normalcy after recovering from COVID-19 to ensure they can resume their normal work activities.

Based on initial interviews, information on people recovering from COVID-19 in Nakhon Pathom over the past 3‐4 years indicates that no agency has monitored the symptoms of those who have recovered, tracked their postrecovery lifestyle, or followed up on long COVID symptoms. Additionally, there has been no data collection on vaccination for people who had COVID-19. This group has been informed that they would develop immunity after infection, leading to a lack of follow-up booster vaccinations.

Given this situation, there is a significant opportunity to design a health service system that includes nursing and continuous care for people living with COVID-19 in Nakhon Pathom. This system should leverage health technology, as the majority of the population now has greater access to technology for health promotion and treatment. It should also include plans to support measures for preventing further outbreaks or new epidemics. This approach would address health maintenance, disease prevention, social distancing, and the adoption of new lifestyle behaviors to live safely with COVID-19.

Therefore, the research team recognizes the importance of addressing these issues and has undertaken the development of a work plan for managing nursing systems and continuous care for people living with COVID-19: A Case Study of Nakhon Pathom Province. This plan aims to examine the nursing and continuing care management work plan for individuals living with COVID-19 in Nakhon Pathom Province and to explore the effectiveness of integrated care approaches, including telehealth and community-based services, by improving access to services, enhancing the care, and providing nursing for patients with post-COVID-19 both at home and within the community. To enhance access to services and improve the quality of care and nursing for post-COVID-19 patients at home and in the community, the following steps are necessary: screening: implement self-assessment tools for evaluating the risk and symptoms post-COVID-19 recovery; tracking and tracing: monitor and record follow-up data by community health service units; surveillance system: establish a system for ongoing observation and reporting; and information transfer system: use health technology to ensure seamless data transfer and communication. By implementing this comprehensive plan, the goal is to prepare the community in Nakhon Pathom to live with COVID-19, ensuring high-quality care and support through the effective use of health technology.

### Research Purpose

The purpose of this article is (1) to study the issues, needs, and systems of nursing and continuous care for people living with post-COVID-19 in Nakhon Pathom Province; (2) to design and develop a database system for continuous monitoring and care of individuals with post-COVID-19 in Nakhon Pathom Province; (3) to develop guidelines for the continuous care of patients recovering from post-COVID-19 in the community in Nakhon Pathom Province; and (4) to evaluate the effectiveness of the database system for continuous monitoring and care of individuals with post-COVID-19 in Nakhon Pathom Province.

### Conceptual Framework

The study is guided by 2 key conceptual frameworks that provide a theoretical basis for the research and the development of the health care system. These frameworks were selected based on their relevance to the goals of the study and their ability to provide a comprehensive approach to managing nursing and continuous care for patients with post-COVID-19.

#### Transitional Care or Transaction Model

The Transitional Care or Transaction Model model [5] emphasizes the importance of continuous care during transitions between health care facilities and home. It focuses on ensuring that patients receive consistent, high-quality care throughout the different stages of their recovery. The model includes three stages: (1) pretransition: the period before the patient leaves the hospital, where the focus is on preparing the patient for the transition to home care; (2) midtransition: the actual transition period, where the patient moves from the hospital to home or another care setting; and (3) posttransition: the period after the patient has returned home, where the focus is on ensuring that the patient continues to receive the care they need to recover fully.

Each stage of the transition requires attention to 4 critical factors:

Information: ensuring that accurate and relevant information is provided to both patients and caregivers. This includes providing clear instructions on medication, follow-up appointments, and any other aspects of the patient’s care plan.

Communication: using effective communication strategies to keep all stakeholders informed and involved. This includes regular updates to the patient’s care team, as well as clear communication with the patient and their family.

Support: offering immediate support to patients and their families as they navigate the health care system. This includes providing access to resources such as social workers, financial counselors, and other support services.

Time: allowing sufficient time for each stage of the transition to be completed successfully. This includes ensuring that the patient has enough time to adjust to their new care setting and that any necessary adjustments to their care plan are made promptly.

The continuous care concept for patients with post-COVID-19 also applies. The primary care behavioral health model by Reiter and team [6] includes the following components: access to health information: ensuring patients and providers have access to relevant health data; health management: efficiently managing health care processes and resources; health coordination: coordinating care among different providers and services; teamwork: facilitating collaborative efforts among health care teams; budget responsibility: managing financial resources effectively; supporting health information technology: using technology to support and enhance health information; and service quality and safety: ensuring high standards of care and safety for patients.

The researchers analyzed, connected, and supported the 2 conceptual frameworks within the context of Nakhon Pathom Province, leading to the design of a database system for data transfer, management, and continuous care for individuals with COVID-19 in the area. The research process involved surveying the situation, issues, and needs of the nursing and continuous care system from the perspectives of professional nurses, public health officials, and patients with post-COVID-19, as well as the general public. This resulted in a practical database system for data transfer, management, and continuous care, which includes the following: information: providing accurate and useful data; communication: using effective communication methods; support: offering timely support as needed; and time: ensuring appropriate and sufficient time for processes. The system addresses health management, coordination, teamwork, and budget responsibility, aiming to ensure safety in daily life.

## Methods

### Study Design

This research uses a research and development approach with 3 stages.

#### Stage 1

Study the problems and needs: this stage investigates the current issues and needs of the nursing and continuous care system for individuals with post-COVID-19 in Nakhon Pathom Province.

#### Stage 2

Design, develop, and test: this stage is to design and develop the database system for continuous monitoring and care of individuals with post-COVID-19 in Nakhon Pathom Province. Subsequently, we synthesized the data from the system to create guidelines for continuous care for patients with post-COVID-19 in community settings within the province.

#### Stage 3

Evaluate the effectiveness: we aimed to assess the effectiveness of the database system for continuous monitoring and care of individuals with post-COVID-19 in Nakhon Pathom Province.

#### Research Area

The research area is Nakhon Pathom Province.

### Population

The included population was patients and families who have had COVID-19 within the past year and have recovered for at least 3 months, residing in Nakhon Pathom Subdistrict, Mueang Nakhon Pathom District, Nakhon Pathom

In the Province, there were a total of 15,117 people. Among them, professional nurses, public health officials, and directors of the community health promotion hospitals in the Mueang District area of Nakhon Pathom Province were included, totaling 50 people.

### Sampling

The sample group was selected using Krejcie and Morgan’s (1970) formula with a 5% margin of error from the total population. For patients and families who have had COVID-19, the sample size is 375 people. For professional nurses, public health officials, and directors of community health promotion hospitals in the Mueang District of Nakhon Pathom Province, all 50 individuals were selected.

### Tools of Research

This study used questionnaires as the primary data collection tool and adopted both qualitative and quantitative methods:

Stage 1 used qualitative tools, which include focus group guidelines; interview guidelines; questionnaire for note-taking; and analysis guidelines and participatory observation.

Stage 2 used the findings from stage 1 to develop a database system for continuous monitoring and care of individuals with post-COVID-19 in Nakhon Pathom Province, which includes system design. We also developed a data management system that collects and stores information on computers and through internet-based databases. Moreover, for data synthesis, we applied matrix-based comparative analysis principles to synthesize the collected data; and created guidelines using the synthesized data to establish guidelines for continuous care of patients with post-COVID-19 in community settings within Nakhon Pathom Province.

Stage 3 involves evaluating the effectiveness of the database system for continuous monitoring and care of individuals with post-COVID-19 in Nakhon Pathom Province. This stage uses quantitative tools by creating a custom questionnaire: developing a specifically designed questionnaire to assess the effectiveness of the database system. This tool will gather quantitative data on how well the system performs in terms of functionality, usability, and overall impact on patient care and management.

The quantitative tool used is a questionnaire designed for data collection, which underwent review by experts in various fields: nursing experts, continuous care nursing experts, epidemic nursing experts, and database system development experts. A total of 3 experts reviewed the questionnaire for content validity, resulting in an Index of Item-Objective Congruence of 0.98. Following the revision of the content based on their feedback, the revised tool was tested for reliability with a sample group of 30 individuals similar to the actual target sample. The reliability analysis of the questionnaire, specifically designed for patients who have had COVID-19 and their families, yielded a content validity index of 0.91.

Qualitative tools included note-taking forms, analysis guides, participatory observation, focus group and interview guidelines, and workshop protocols. Content validity was confirmed by experts, with a Content Validity Index of 0.90 for the nurse or public health questionnaire and 1.00 for interviews with hospital directors, ensuring tool reliability.

### Data Collection

Data were collected with 2 years of follow-up in 3 steps as follows.

#### Step 1

Step 1 involves studying the problems and needs of the nursing and continuous care system for individuals with COVID-19 in Nakhon Pathom Province. The focus group involves examining the situation, identifying issues, and determining requirements from a total of 375 participants. The qualitative data are synthesized to create a comprehensive map of the issues and needs of the nursing and continuous care system for individuals with COVID-19 in Nakhon Pathom Province. The summarized results will inform and guide the development process in step 2.

#### Step 2

In step 2, the researchers designed a database system for continuous monitoring and care of individuals with COVID-19 in Nakhon Pathom Province using data from step 1. This involved developing the system with technology through 4 datasets: information, communication, support, and time. The goal is for the community health promotion hospitals in Nakhon Pathom to use the database system for data transfer, management planning, and continuous care, enabling individuals in Mueang District, Nakhon Pathom Province, to lead live safe and stable lives while managing their condition. The system design also incorporated measures from the Personal Data Protection Act.

Test and pilot the database system: Tests and trial runs of the database system will be conducted for continuous monitoring and care of individuals with COVID-19 in Nakhon Pathom Province. This involves evaluating the system’s functionality, usability, and effectiveness in real-world scenarios to ensure it meets the needs of managing and supporting the affected population.

Develop guidelines for continuous care: The researchers used the analysis results from step 1, combined with relevant data from additional research, to create guidelines for the continuous care of patients with post-COVID-19 in community settings within Nakhon Pathom Province. This involved synthesizing the information to establish practical and effective care practices tailored to the needs of the local population.

#### Step 3

Step 3 involves evaluating the effectiveness of the database system for continuous monitoring and care of individuals with COVID-19 in Nakhon Pathom Province.

The evaluation will involve a sample group consisting of professional nurses, public health officials, and Directors of Community Health Promotion Hospitals. In total, 50 individuals from the Mueang District of Nakhon Pathom Province will be involved in assessing the system’s performance and effectiveness.

Summarize the effectiveness of the database system: The data on the performance and effectiveness of the database system for continuous monitoring and care of individuals with COVID-19 in Nakhon Pathom Province will be compiled and analyzed. This includes evaluating how well the system meets its objectives and supports patient management.

Develop recommendations and user manual: Recommendations will be provided based on the evaluation findings and a comprehensive user manual for the database system will be prepared. The manual will guide users on how to effectively operate the system and integrate it into their continuous care practices for individuals with COVID-19 in Nakhon Pathom Province.

### Ethical Considerations

This research has received ethical approval from the Human Research Ethics Committee of Nakhon Pathom Rajabhat University, with approval numbers 047/2565-047/2566. Participants in the research are free to withdraw from providing information or participating in activities at any time during the data collection process. They can also withdraw from the research process altogether if they are uncomfortable with using the data or engaging in the activities.

### Data Analysis

In steps 1 and 2, the analysis focuses on understanding the problems, needs, and continuous care system for individuals with COVID-19 in Nakhon Pathom Province. This involves process analysis, content comparison, interpretation, summarization, transcription, categorization, synthesis of sentences, and validation: triangulating data by cross-checking findings from multiple sources and methods to ensure accuracy and reliability. This approach ensures a comprehensive and reliable analysis of qualitative data.

In step 3, which involves evaluating the effectiveness of the database system for continuous care of individuals with COVID-19 in Nakhon Pathom Province, the analysis focuses on descriptive statistics, percentages, frequency distribution, mean and SD. These statistical methods help summarize and interpret the quantitative data collected during the evaluation of the database system, providing insights into its effectiveness and performance.

## Results

### Phase 1: The Problems and Needs of the Nursing and Continuous Care System for Individuals With COVID-19 in Nakhon Pathom Province

The results of phase 1 revealed 5 key issues regarding the health care system and continuous care needs for the public living with COVID-19 in Nakhon Pathom province.

#### Issues With Health Care Service

When the rapid antigen test results are positive, patients receive management to enter the health care system according to the hospital’s primary health care promotion unit in the area they reside. However, some individuals do not access the service system, instead focusing on self-care using principles of isolation from family members and primarily treating themselves with herbal remedies.

“Long wait times for service, being ill without income, recovered but symptoms persist.” After recovering, issues arise due to reflections that the patient group accessing hospital services receives care at the provincial hospital, undergoes hospital checks after staying at the field hospital for 10 days, then returns home for 4 days of continued care at the local health promotion hospital via line group reporting and community health volunteers. Friends and neighbors help report symptoms through line groups and report complications or additional symptoms, with the hospital sending medication to take.

To address the needs for care and nursing following recovery from COVID-19, the following steps are recommended:

Facility preparation: Local service facilities should prepare beds for bedridden patients or elderly individuals recovering from COVID-19. This includes setting up systems for meal delivery in the area to facilitate convenience during isolation.

Continued follow-up: There should be continuous monitoring and follow-up after recovery from COVID-19. It is crucial to advise individuals to maintain their health and provide education on self-care, particularly regarding monitoring symptoms such as fatigue and shortness of breath post-COVID.

Vaccination guidance: Guidance should be provided regarding vaccination. It’s important to emphasize that vaccination against COVID-19 and influenza can strengthen immunity and prevent re-infection with COVID-19. This also reduces the risk of transmitting the virus again in the community.

These measures aim to ensure comprehensive care and support for individuals postrecovery, promoting their well-being and reducing the likelihood of recurrence or complications related to COVID-19.

The issue of providing health information and preparing health data for patients with COVID-19 should be led by health care professionals in the area, especially professional nurses, public health officials, and community health volunteers. They should serve as information leaders for self-care among patients with COVID-19. Communication channels should emphasize awareness through voice messaging systems and community outreach via TV, Facebook, and the internet. Information should be obtained from local health authorities, neighbors, family members, hospitals, and local health promotion hospitals. This ensures that individuals receive clear and adequate information on self-care and COVID-19 prevention efforts.

Communication strategies appropriate during various stages—awareness, illness, recovery, and reintegration into society—include discreet doctor notification via phone to contact health services upon symptoms or diagnosis. During illness, nurses may inquire daily for the initial treatment group, approximately 10‐15 days, while those self-treating with family monitor health, consumption, and home isolation postrecovery. Initially worried about social acceptance post-COVID, more recently adjusting, radio communication shares self-care after diagnosis and primary care treatments

The key support factors during illness, recovery, and postreintegration into society include increased family caregiving, mutual encouragement among friends, and involvement from doctors, nurses, and relevant officials. Family members, community health volunteers, and local health authority representatives play pivotal roles in communication and primary care. Spouses, children, and extended family members provide assistance during isolation, particularly for COVID-19 cases or households with elderly members requiring separate care, ensuring continuous support.

The issue concerning the timeframe after recovering from COVID-19 spans approximately 1 week to 6 months, with varying durations of symptoms for each individual. Some may experience symptoms for up to 15 days, isolating from household members for about 10‐15 days due to fear of social stigma and concern about others’ perceptions of ongoing illness. They separate their food and personal items, use a separate room for rest, meals, and medication, and gradually return to normal life after recovery. However, lingering symptoms such as cough, fatigue, and underlying health conditions persist.

### Phase 2 Outcomes: Development and Design of a Database System for Continuous Monitoring and Care for Residents Living With COVID-19 in Nakhon Pathom Province, Specifically for Patients With Post-COVID-19 in Community Settings

#### Dataset for Developing a Continuous Monitoring and Care Database System for Residents Living With COVID-19

Part 1: General information, which includes household member general information, information related to household head, characteristics of occupations, household security information, household environmental management information, health information, and communication information.

Part 2: Continuous care management data, which include chronic diseases, persistent symptoms after recovering from COVID-19 1 year later, and health care guidelines for the population after recovering from COVID-19.

Part 3: Management outcome data. Analyzing data from parts 1 and 2 to analyze the outcomes resulting from comprehensive management efforts as follows:

CODE_PC1 : Seeking health knowledge and management post-COVID-19
CODE_PC2 : Risk control and safety post-COVID-19 by telenursing
CODE_PC3: Self-care post-COVID-19
CODE_PC4: Self-control psychological health post-COVID-19
CODE_PC5: Satisfaction with referral care post-COVID-19

### Health Care Guidelines for the Population After Recovering From COVID-19

#### Guidelines for Self-Care

Using digital resources related to COVID-19 includes recommending the use of apps, websites, and information from health promotion hospitals to provide information on COVID- 19 infection; recommend vaccination information; and advise on using apps and websites related to COVID-19 for village health volunteers.

#### Guidelines for Managing COVID-19 Remotely Through Telenursing Systems

These guidelines include the following: providing consultation and advice via phone by community health promotion hospitals; organizing remote educational activities on self-protection, hand hygiene, wearing masks, and updated information on COVID-19 for patients, families, and communities; conducting remote disease management and nursing activities by health care experts; and assessing and screening post-COVID symptoms, interpreting rapid antigen test results, managing medications, and monitoring respiratory symptoms.

#### Guidelines for Providing Post-COVID-19 Physical Health Care Services

These guidelines include the following: annual health check-ups, particularly chest X-rays and disease screening; home visits by community health volunteers; remote home care services by community nurses or public health professionals; providing advice on self-care through purchasing medications and treatments from pharmacies, medical clinics, and nursing clinics in the area; and organizing campaigns to encourage vaccination against COVID-19 and influenza.

#### Guidelines for Providing Mental Health Services Postrecovery From COVID-19

These guidlines are used for providing services to assess and screen for depression and offering mental health counseling through hotline services.

#### Continuous Care Guidelines for Referral in Case of Emergencies Postrecovery From COVID-19

These guidelines include the following: arranging services for severe symptoms or emergencies providing remote nursing services for continuous treatment information dissemination.

The outcome is the database of results from the continuous care management of COVID-19 infection 1 year postrecovery.

### Phase 3: Evaluate the Effectiveness of the Database System for Continuous Monitoring and Care of Individuals With Post-COVID-19 in Nakhon Pathom Province

The researchers implemented a database system for continuous monitoring and care for the public affected by COVID-19 in Nakhon Pathom Province. They evaluated its effectiveness among 50 health care professionals, including registered nurses, public health officers, and directors of sub-district health promotion hospitals in Mueang District, Nakhon Pathom Province. The overall effectiveness was found to be at the highest level (mean 4.51, SD 0.59), with the highest performance specifically in data security (mean 4.59, SD 0.57). Following closely was the capability to perform tasks effectively (mean 4.53, (SD 0.56).

## Discussion

### Principal Findings

This study aimed to develop a nursing and continuous care management work plan for individuals living with COVID-19 in Nakhon Pathom Province, focusing on identifying needs, designing an integrated database system, and evaluating its effectiveness. The findings highlight significant challenges in the health care service system, including limited access to care, reliance on self-treatment, and the persistence of post-COVID-19 symptoms. In response, a comprehensive care model was designed to address gaps in service delivery through the use of digital technologies, community-based care strategies, and structured follow-up mechanisms. The final phase demonstrated the system’s high level of effectiveness in practice, particularly in data security and task performance, as evaluated by health care professionals. Results showed high overall effectiveness (mean 4.51, SD 0.59), with data security rated highest (mean 4.59, SD 0.57), followed by task performance (mean 4.53, SD 0.56). This confirmed the system’s capacity to support sustainable, technology-enabled post-COVID care at the community level.

Based on the situation of health care and continuous care systems for the public living with COVID-19 in Nakhon Pathom Province, it was found that symptoms persist approximately 6 months postinfection. According to Davis et al [7] for the majority of respondents (>91%), the time to recovery exceeded 35 weeks. Common post-COVID symptoms include persistent cough and fatigue, consistent with studies by Thekgungsakdakul et al [8] and Ruengsong et al [9]. However, other research suggests that patients with COVID-19 may have an increased risk of developing respiratory diseases, and the risk increases with the severity of infection and reinfection [10].

There is also evidence of anxiety and social withdrawal, echoing findings by Tongtaeng and Sisawang [11], which highlight significant psychological impacts such as depression that necessitate evaluation following COVID-19 infection management protocols [12]. This research defines self-management outcomes in mental health post-COVID symptom onset.

From the study findings, it was observed that patients experiencing post-COVID symptoms have reduced access to treatment services at local facilities and increasingly rely on telehealth systems for care. They still require support from professional nurses and community health volunteers for continuous care. Consequently, the research analysis proposes health care guidelines for the public following recovery from COVID-19, consisting of five key strategies: (1) self-care guidance through digital information access related to COVID-19; (2) management of COVID-19 via telehealth nursing systems; (3) physical health care services postrecovery; (4) mental health care services postrecovery; and (5) continuous care guidelines for referral cases reported after recovery. The integration of telehealth systems has played a critical role in enhancing nursing care during the COVID-19 pandemic. Prior studies have demonstrated that telemedicine not only supports effective monitoring of patients with COVID-19 while minimizing transmission risks but also contributes to the continuity and quality of care delivery [13,14].

These align with the findings of Thongnopakun et al’s [15] study on knowledge and attitudes towards patient follow-up among community health volunteers before and after training, indicating significant differences. They also follow the guidelines of the Medical Research and Technology Evaluation Institute [16], emphasizing self-care and receiving services from local health units or seeking telemedicine advice if symptoms persist beyond 2 months to consult specialized physicians for developing health care strategies for patients experiencing post-COVID symptoms, including the studies of Chugamnern et al [17], which emphasize the importance of evaluating data from patients’ families and communities, thereby reflecting the importance and necessity of designing a continuous care tracking database system for the public living with COVID-19 to improve service provision in the future.

### Suggestions

A network for emergency care or urgent assistance among local volunteers and the health service network to prevent diseases should be established.

Health service units should establish policies on data management for health service leaders, communication systems to develop a continuous care system focusing on home visits, and support management to care for patients with post-COVID or patients with other epidemic diseases.
